# A Bayesian Network View on Nested Effects Models

**DOI:** 10.1155/2009/195272

**Published:** 2008-11-12

**Authors:** Cordula Zeller, Holger Fröhlich, Achim Tresch

**Affiliations:** 1 Department of Mathematics, Johannes Gutenberg University, 55099 Mainz, Germany; 2 Division of Molecular Genome Analysis, German Cancer Research Center, 69120 Heidelberg, Germany; 3 Gene Center, Ludwig Maximilians University, 81377 Munich, Germany

## Abstract

Nested effects models (NEMs) are a class of probabilistic models that were designed to reconstruct a hidden signalling structure from a large set of observable effects caused by active interventions into the signalling pathway. We give a more flexible formulation of NEMs in the language of Bayesian networks. Our framework constitutes a natural generalization of the original NEM model, since it explicitly states the assumptions that are tacitly underlying the original version. Our approach gives rise to new learning methods for NEMs, which have been implemented in the /*Bioconductor* package nem. We validate these methods in a simulation study and apply them to a synthetic lethality dataset in yeast.

## 1. Introduction

Nested effects models (NEMs) are a class of probabilistic models. They aim to reconstruct a hidden signalling structure (e.g., a gene regulatory system) by the analysis of high-dimensional phenotypes (e.g., gene expression profiles) which are consequences of well-defined perturbations of the system (e.g., RNA interference). NEMs have been introduced by Markowetz et al. [1], and they have been extended by Fröhlich et al. [2] and Tresch and Markowetz [3], see also the review of Markowetz and Spang [4]. There is an open-source software package "*nem*" available on the platform /*Bioconductor* [5, 13], which implements a collection of methods for learning NEMs from experimental data. The utility of NEMs has been shown in several biological applications (*Drosophila melanogaster* [1], *Saccharomyces cerevisiae* [6], estrogen receptor pathway, [7]). The model in its original formulation suffers from some ad hoc restrictions which seemingly are only imposed for the sake of computability. The present paper gives an NEM formulation in the context of Bayesian networks (BNs). Doing so, we provide a motivation for these restrictions by explicitly stating prior assumptions that are inherent to the original formulation. This leads to a natural and meaningful generalization of the NEM model.

The paper is organized as follows. Section 2 briefly recalls the original formulation of NEMs. Section 3 defines NEMs as a special instance of Bayesian networks. In Section 4, we show that this definition is equivalent to the original one if we impose suitable structural constraints. Section 5 exploits the BN framework to shed light onto the learning problem for NEMs. We propose a new approach to parameter learning, and we introduce structure priors that lead to the classical NEM as a limit case. In Section 6, a simulation study compares the performance of our approach to other implementations. Section 7 provides an application of NEMs to synthetic lethality data. In Section 8, we conclude with an outlook on further issues in NEM learning.

## 2. The Classical Formulation of Nested Effects Models

**Figure 1:**
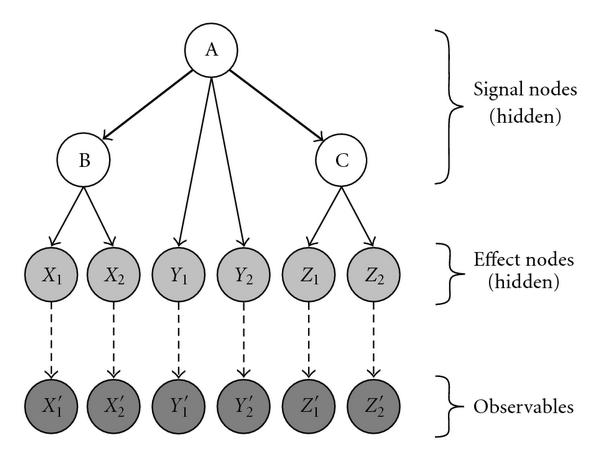
**Example of a Nested effects model in its Bayesian network formulation.** The bold arrows determine the graph , the solid thin arrows encode . Dashed arrows connect the effects to their reporters.

For the sake of self-containedness, we briefly recall the idea and the original definition of NEMs, as given in [3]. NEMs are models that primarily intend to establish causal relations between a set of binary variables, the signals . The signals are not observed directly rather than through their consequences on another set of binary variables, the effects . A variable assuming the value , respectively,  is called *active*, respectively, *inactive*. NEMs deterministically predict the states of the effects, given the states of the signals. Furthermore, they provide a probabilistic model for relating the predicted state of an effect to its measurements. NEMs consist of a directed graph  the nodes of which are the variables . Edges represent dependencies between their adjacent nodes. An arrow pointing from  to  means that  is active whenever  is active. To be more precise, the graph  can be decomposed into a graph , which encodes the information flow between the signals, and a graph  which relates each effect to exactly one signal, see Figure 1. The effects that are active as a consequence of a signal  are those effects that can be reached from  via at most one step in , followed by one step in . Let  denote the predicted state of  when signal  is activated, and let  be the matrix of all predicted effects.

For the probabilistic part of the model, let  be the data observed at effect  when signal  is activated (which, by the way, need not be binary and may comprise replicate measurements), and let  be the matrix of all measurements. The stochastic model that relates the predictions  to the experimental data  is given by a set of "local" probabilities . There are several ways of specifying , depending on the kind of data and the estimation approach one wants to pursue (see [1–3]). An NEM is completely parameterized by  and , and, assuming data independence, its likelihood is given by(1)

## 3. The Bayesian Network Formulation of Nested Effects Models

A Bayesian network describes the joint probability distribution of a finite family of random variables (the nodes) by a directed acyclic graph  and by a family of local probability distributions, which we assume to be parameterized by a set of parameters  (for details, see, e.g., [8]). We want to cast the situation of Section 2 in the language of Bayesian networks. Assuming the acyclicity of the graph  of the previous section, this is fairly easy. A discussion on how to proceed when  contains cycles is given in Section 4. We have to model a deterministic signalling hierarchy, in which some components  can be probed by measurements, and some components  are perturbed in order to measure the reaction of the system as a whole. All these components  will be *hidden* nodes in the sense that no observations will be available for , and we let the topology between these nodes be identical to that in the classical model. In order to account for the data, we introduce an additional layer of observable variables (*observables*, ) in an obvious way: each effect node  has an edge pointing to a unique (its) observable node  (see Figure 1). Hence, , and we call *the observation of*.

Let  be the set of parents of a node , that is, the set of nodes that are direct predecessors of . For notational convenience, we add a zero node , , which has no parents, and which is a parent of all hidden nodes (but not of the observables). Note that by construction,  is not empty unless  is the zero node. For the hidden nodes, let the local probabilities describe a deterministic relationship,(2)

We slightly abuse notation by writing  for the maximum value that is assumed by a node in . Obviously, all hidden nodes are set to 0 or 1 deterministically, given their parents. The local probabilities , , remain arbitrary for the moment. Assume that we have made an intervention into the system by activating a set of nodes . This amounts to cutting all edges that lead to the nodes in  and setting their states to value 1. When an intervention  is performed, let  be the value of . This value is uniquely determined by , as the next lemma shows.

Lemma 3.1. 

 if and only if  can be reached from one of the nodes in  by a directed path in  (i.e., there exists a sequence of directed edges in  possibly of length zero, that links an  to ). When performing an intervention , we, therefore, have (3)

Proof. 

The proof is straightforward though somewhat technical and may be skipped for first reading. Let  be an ordering of the nodes compatible with , which means , . Such an ordering exists because the graph connecting the states is acyclic. The proof is by induction on the order, the case  being trivial. If , there is nothing to prove. Hence, we may assume  in the graph which arises from  by cutting all edges that lead to a node in . Since , it follows that  if and only if  for some . This holds exactly if  for some  (in particular, ). By induction, this is the case if and only if there exists an  and a directed path from  to , which can then be extended to a path from  to .

Let  be an observation of the effects generated during intervention . Marginalization over the hidden nodes yields(4)

Since by (3) there is only one possible configuration for the hidden nodes, namely, , , (4) simplifies to(5)(6)

This formula is very intuitive. It says that if an intervention  has been performed, one has to determine the unique current state of each effect node. This, in turn, determines the (conditional) probability distribution of the corresponding observable node, for which one has to calculate the probability of observing the data. The product over all effects then gives the desired result.

## 4. Specialization to the Original NEM Formulation

In fact, (6) can be written as(7)

Let , , and . Following the NEM formulation of [3], we consider all replicate measurements of an intervention  as generated from its own Bayesian network, and we try to learn the ratio  separately for each intervention . Therefore, we include  into the subscript. Taking logs in (7), it follows that(8)

Suppose that we have performed a series  of interventions, and we have generated observations , respectively. Assuming observational independence, we get(9)

with the matrices  and . The importance of (9) lies in the fact that it completely separates the estimation steps for  and . The information about the topology  of the Bayesian network enters the formula merely in the shape of , and the local probability distributions alone define . Hence, prior to learning the topology, one needs to learn the local probabilities only for once. Then, finding a Bayesian network that fits the data well means finding a topology which maximizes .

In the original formulation of NEMs, it is assumed that the set of interventions equals the set of all single-node interventions, , . As pointed out in Section 2, the topology of the BN can be captured by two graphs  and , which we identify with their corresponding adjacency matrices  and  by abuse of notation. The  adjacency matrix  describes the connections among signals, and the  adjacency matrix  encodes the connection between signals and effects. For convenience, let the diagonal elements of  equal . Denote by  the adjacency matrix of the transitive closure of . Check that by Lemma 3.1, . Therefore, we seek (10)

which for transitively closed graphs  is exactly the formulation in [3]. It has the advantage that given , the optimal  can be calculated exactly and very fast, which dramatically reduces the search space and simplifies the search for a good graph . The BN formulation of NEMs implies via (10) that two graphs  are indistinguishable (likelihood equivalent, they fit all data equally well) if they have the same transitive closure. It is a subject of discussion whether the transitive closure of the underlying graph is a desirable property of such a model (think of causal chains which are observed in a stable state) or not (think of the dampening of a signal when passed from one node to another, or of a snapshot of the system where the signalling happens with large time lags), see [9].

It should be mentioned that the graph topology in our BN formulation of NEMs is necessarily acyclic, whereas the original formulation admits arbitrary graphs. This is only an apparent restriction. Due to the transitivity assumption, effects that connect to a cycle of signals will always react in the same way. This behaviour can also be obtained by arranging the nodes of the cycle in a chain and connecting the effects to the last node of the chain. This even leaves the possibility for connecting other effects to only a subset of the signals in the cycle by attaching them to a node higher up in the chain. As a consequence, admitting cycles does not extend the model class of NEMs in the Bayesian setting.

Although the original NEM model is algebraically and computationally appealing, it has some drawbacks. Learning the ratio  separately for each intervention  entails various problems as follows.

(1) Given an observation  at observable  together with the state of its parent , the quantity  should not depend on the intervention  during which the data were obtained, by the defining property of Bayesian networks. However, we learn the ratio  separately for each intervention, that is, we learn separate local parameters , which is counterintuitive.

(2) Reference measurements  are used to calculate the ratio , raising the need for a "null" experiment corresponding to an unperturbed observation  of the system, which might not be available. The null experiment enters the estimation of each ratio . This introduces an unnecessary asymmetry in the importance of intervention  relative to the other interventions.

(3) The procedure uses the data inefficiently since for a given topology, the quantities of interest , respectively,  could be learned from *all* interventions that imply , respectively, , providing a broader basis for the estimation.

The method proposed in the last item is much more time-consuming, since the occurring probabilities have to be estimated individually for each topology. However, such a model promises to better capture the real situation, so we develop the theory into this direction.

## 5. NEM Learning in the Bayesian Network Setting

Bear in mind that a Bayesian network is parameterized by its topology  and its local probability distributions, which we assume to be given by a set of local parameters . The ultimate goal is to maximize . In the presence of prior knowledge, (we assume independent priors for the topology and the local parameters), we can write(11)

from which it follows that(12)

If it is possible to solve the integral in (12) analytically, it can then be used by standard optimization algorithms for the approximation of . This full Bayesian approach will be pursued in Section 5.1. If the expression in (12) is computationally intractable or slow, we resort to a simultaneous maximum a posteriori estimation of  and , that is,(13)

The hope is that the maximization  in (13) can be calculated analytically or at least very efficiently, see [3]. Then, maximization over  is again done using standard optimization algorithms. Section 5.2 is devoted to this approach.

### 5.1. Bayesian Learning of the Local Parameters

Let the topology  and the interventions  be given. Let  denote the number of times the observable  was reported to take the value , while its true value was , and let  be the number of measurements taken from  when its true value is :(14)

Binary Observables

The full Bayesian approach in a multinomial setting was introduced by Cooper and Herskovits [10].

The priors are assumed to follow beta distributions:(15)

Here, , and  are shape parameters, which, for the sake of simplicity, are set to the same value for every effect . This assumption can be easily dropped and different priors may be used for each effect.

In this special setting with binomial nodes with one parent, the well-known formula of Cooper and Herskovitz can be simplified to(16)

Continuous Observables

Let us assume  to be normally distributed with mean  and variance , , . We refer to the work of Neapolitan [8] for the calculation of this section. Let the prior for the precision  follow a Gamma distribution,(17)

Given the precision , let the conditional prior for the mean  be(18)

So the Data of observable  given its parent's state  is(19)

Then,(20)

The data enters this equation via(21)

### 5.2. Maximum Likelihood Learning of the Local Parameters

Let the topology  and the interventions  be given. For learning the parameters of the local distributions , we perform maximum likelihood estimation in two different settings. The observables are assumed to follow either a binomial distribution or a Gaussian distribution.

Binary Observables

For an effect , let its observable  be a binary random variable with values in , and let , . The model is then completely parameterized by the topology  and .

Note that(22)

with . The parameter set  that maximizes expression (22) is(23)

(the ratios with a denominator of zero are irrelevant for the evaluation of (22) and are set to zero).

Continuous Observables

There is an analogous way of doing ML estimation in the case of continuous observable variables if one assumes  to be a normal distribution with mean  and variance , , .

Note that(24)

with(25)

The parameter set  maximizing expression (24) is(26)

(quotients with a denominator of zero are again irrelevant for the evaluation of (24) and are set to zero). Note that in both the discrete and the continuous case,  depends on the topology , since the topology determines the values of , , .

### 5.3. Structure Learning

It is a major achievement of NEMs to restrict the topology of the underlying graphical structure in a sensible yet highly efficient way, thus, tremendously reducing the size of the search space. There is an arbitrary "core" network consisting of signal nodes, and there is a very sparse "marginal" network connecting the signals to the effects. It is, however, by no means necessary that the core network and the signal nodes coincide. We propose another partition of the hidden nodes into core nodes  and marginal nodes , , which may be distinct from the partition into signals and effects, . No restrictions are imposed on the subgraph generated by the core nodes (except that the graph has to be acyclic). The key semantics of NEMs is that marginal nodes are viewed as the terminal nodes of a signalling cascade. The requirement that the marginal nodes have only few or at most one incoming edge can be translated into a well-known structure prior  (see, e.g., [12]) which penalizes the number of parents of marginal nodes:(27)

For the penalty parameter , this is the original NEM restriction. If , each marginal node can be assigned to all suitable core nodes. As a consequence, there is always a best scoring topology with an empty core graph.  makes signalling to the marginal nodes "expensive" relative to signalling in the core graph. It is unclear how to choose  optimally, so we stick to the choice  for the applications. Simulation studies have shown that a simple gradient ascent algorithm does very well in optimizing the topology of the Bayesian network, compared to other methods that have been proposed [7].

## 6. Simulation

### 6.1. Network and Data Sampling

The ML and the Bayesian method for parameter learning have been implemented in the *nem* software [13], which is freely available at the /*Bioconductor* software platform [5]. To test the performance of our method, we conducted simulations with randomly created acyclic networks with  signals. The out-degree  of each signal was sampled from the power-law distribution(28)

where  is an appropriate normalization constant. Binary data (1 = effect, 0 = no effect) was simulated for the perturbation of each signal in the created network using 4 replicate measurements with type-I and type-II error rates  and , which were drawn uniformly from  and  for each perturbation separately. This simulates individual measurement error characteristics for each experiment.

### 6.2. Results

**Figure 2:**
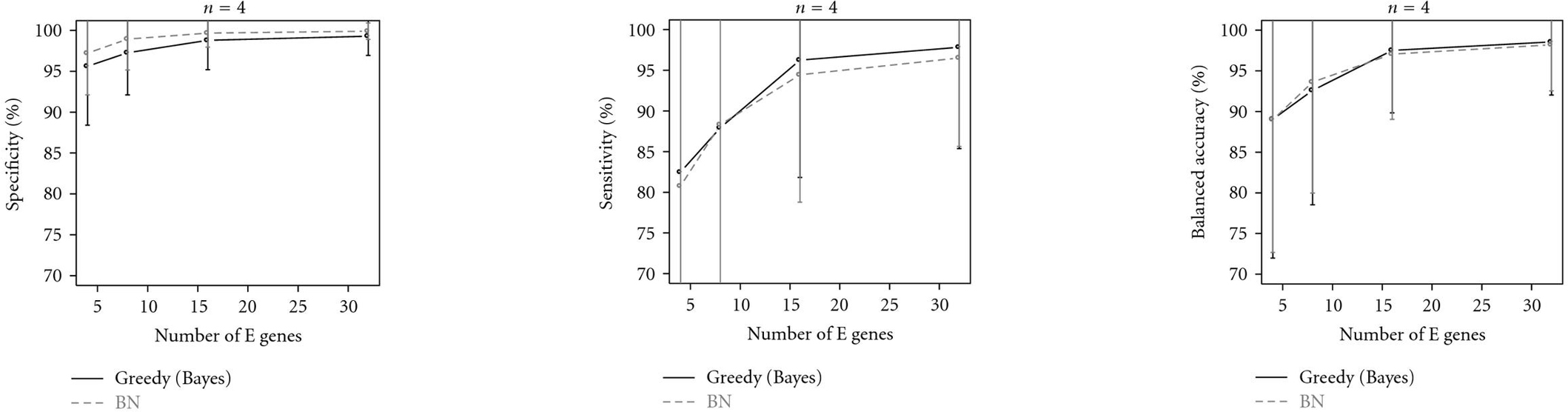
**Results (specificity, sensitivity, and balanced accuracy) of simulation run.** The continuous line (greedy (Bayes)) describes the performance of the traditional NEM method, the dashed line stands for our new approach via Bayesian networks.

We compared our Bayesian network model with the classical NEM using a greedy hill-climbing algorithm to find the best fitting connection between signals. We simulated  and 250 effect nodes, and for each number of effects, 100 random networks were created as described above. Figure 2 demonstrates that both approaches perform very similarly.

## 7. Application

**Figure 3:**
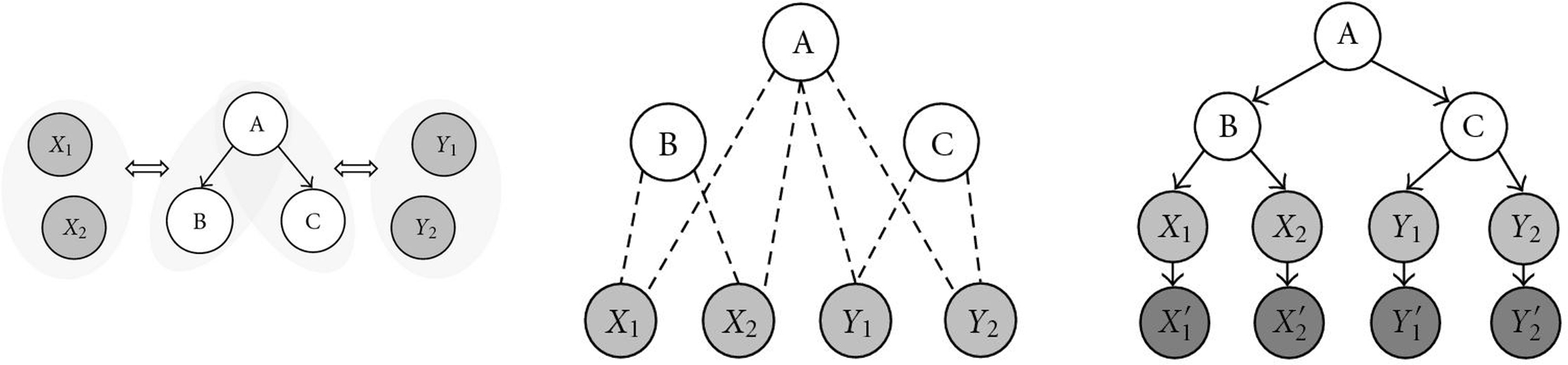
**Schematic reconstruction of a signalling pathway through synthetic lethality data.** (a) A situation in which there are two pairs of complementary pathways ( and ). (b) Model of the situation as follows: the primary knockouts are considered signals  (they are not observed). As those are our genes of interest, they will also form the core nodes. The secondary effects are accessible to observation and, therefore, represented by the effects , and . Each SL pair is connected by a dashed line. (c) NEMs that might be estimated from (b), using binary observables and one of the approaches in Sections 5.1 or 5.2.

We apply the BN formulation of the NEM methodology to a dataset of synthetic lethality interactions in yeast. We reveal hierarchical dependencies of protein interactions. Synthetic lethality (SL) is the phenomenon that a cell survives the single gene deletion of a gene A and a gene B, but the double deletion of A and B is detrimental. In this case, A and B are called SL partners or an SL pair. It has been shown in [11] that it is not so much SL partners themselves whose gene products participate in the same protein complex or pathway, rather than genes that *share* many SL partners. The detection of genetic interactions via synthetic lethality screens and appropriate computational tools is a current area of research, see [14]. Ye and Peyser define a hypergeometric score function to test whether two genes have many SL partners in common. They apply their methodology to a large SL data set [15] for finding pairs (and, consequently, clusters) of genes whose products are likely to participate in the same pathway. We extend their approach as explained in Figure 3. SL partnership arises (not exclusively, but prevalently) among genes pertaining to two distinct pathways that complement each other in a vital cell function. If a gene A is upstream of gene B in some pathway, a deletion of gene A will affect at least as many pathways as a deletion of gene B. Hypothesizing a very simplistic world, all SL partners of B will as well be SL partners of A; but this subset relation can be detected by NEMs. Take the primary knockout genes as core nodes, and the secondary knockout genes as marginal nodes, which are active given a primary knockout whenever SL occurs. We used the dataset from [15] and chose 40 primary knockout genes having the most SL interaction partners as core genes, and included all their 194 SL partners as marginal nodes. An NEM with binary observables was estimated, both with the maximum likelihood approach and in the Bayesian setting. It should be emphasized that NEM estimation for this dataset is only possible in the new BN setting because there is no canonical "null experiment," which enables us to estimate the likelihood ratios  needed in the classical setting in (7), (8), [14].

**Figure 4:**
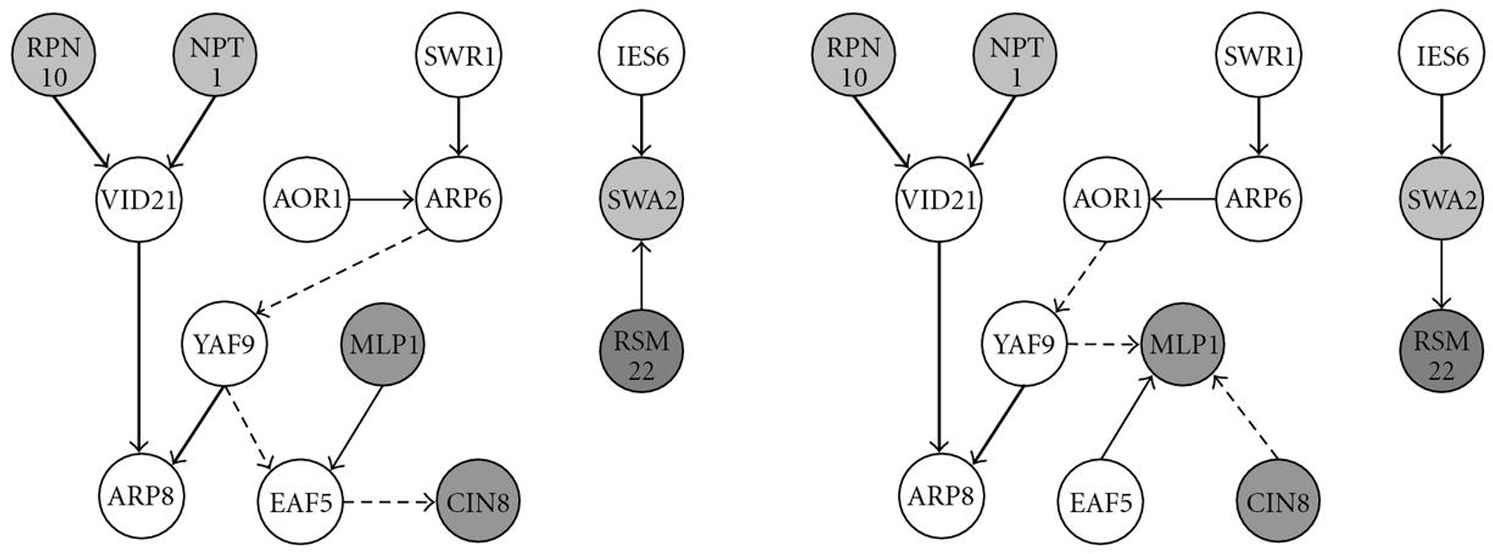
**NEMs constructed from the SL data.** Only core genes that have at least one edge are shown. (a) The ML estimate. (b) The Bayesian estimate (the prior choice (see (15)) was , respectively, ). Nodes with the same shading pertain to the same clusters that were defined by Ye et al. [11]. Bold arrows appear in both reconstructions, thin arrows reverse their direction, and dashed arrows are unique to each reconstruction.

Figure 4 displays the results of the NEM reconstruction. The NEMs estimated by both methods agree well as far as the hierarchical organisation of the network is concerned. However, they do not agree well with the clusters found in [11]. We refrain from a biological interpretation of these networks, since the results are of a preliminary nature. In particular, the reconstruction does not take advantage of prior knowledge, and the postulated edges were not validated experimentally.

## 8. Summary and Outlook

Some aspects of the classical NEM concept appear in a different light when stated in the BN framework. Mainly, these are three folds: (1) the learning of the local parameters, for which we proposed new learning rules; (2) the structural constraints, they can be cast as priors on the NEM topology; (3) the distinction between hidden and observable nodes, which can be different from that of core nodes and marginal nodes.

We proposed some new lines of investigation, like a full Bayesian approach for the evaluation of , and a smooth structure prior with continuous penalty parameter . It is much easier to proceed in the BN framework and implement, for example, a boolean logic for the signal transduction, which is less simplistic than in the current model. A straightforward application of NEMs in their BN formulation to synthetic lethality data demonstrated the potential of the NEM method, with the purpose of stimulating further research in that field. 
